# Genome-Wide Identification and Expression Pattern of Cytochrome P450 Genes in the Social Aphid *Pseudoregma bambucicola*

**DOI:** 10.3390/insects14020212

**Published:** 2023-02-20

**Authors:** Jianjun Lu, Hui Zhang, Qing Wang, Xiaolei Huang

**Affiliations:** 1 State Key Laboratory of Ecological Pest Control for Fujian and Taiwan Crops, College of Plant Protection, Fujian Agriculture and Forestry University, Fuzhou 350002, China; 2 Key Laboratory of Zoological Systematics and Evolution, Institute of Zoology, Chinese Academy of Sciences, Beijing 100101, China

**Keywords:** *Pseudoregma bambucicola*, P450, phylogenetic analysis, expression profiling

## Abstract

**Simple Summary:**

Differences in P450 gene composition and expression have important implications for the normal growth and development of insects. *Pseudoregma bambucicola* is a social aphid that produces genetically identical first-instar soldiers and normal nymphs within the colonies. The soldiers have harder epidermis, enlarged forelegs, longer frontal horns, and are sterile. We identified 43 P450 genes in this species and inferred their possible function based on phylogenetic analyses. We identified a number of P450 genes that are expressed at higher levels in soldiers than in normal nymphs and adult aphids, such as *CYP18A1*, *CYP4G332,* and *CYP4G333*. This study provides a basis for further exploring the function of P450 genes in this social insect.

**Abstract:**

Cytochrome P450 monooxygenases (P450s) have a variety of functions, including involvement in the metabolism of exogenous substances and the synthesis and degradation of endogenous substances, which are important for the growth and development of insects. *Pseudoregma bambucicola* is a social aphid that produces genetically identical but morphologically and behaviorally distinct first-instar soldiers and normal nymphs within colonies. In this study, we identified 43 P450 genes based on *P. bambucicola* genome data. Phylogenetic analysis showed that these genes were classified into 4 clans, 13 families, and 23 subfamilies. The CYP3 and CYP4 clans had a somewhat decreased number of genes. In addition, differential gene expression analysis based on transcriptome data showed that several P450 genes, including *CYP18A1*, *CYP4G332,* and *CYP4G333*, showed higher expression levels in soldiers compared to normal nymphs and adult aphids. These genes may be candidates for causing epidermal hardening and developmental arrest in soldiers. This study provides valuable data and lays the foundation for the study of functions of P450 genes in the social aphid *P. bambucicola.*

## 1. Introduction

Cytochrome P450 monooxygenases (P450s) are a large gene superfamily of heme-thiolate proteins. P450s are widely distributed in animals, plants, and microorganisms, as well as in different tissues of the same organism [1]. These enzymes are involved in synthesizing and inactivating endogenous and exogenous substances in insects; therefore, they play many important functional roles in the growth and development of insects [2]. According to the nomenclature of P450s (CYPs), the first number after CYP represents a family, the subsequent letter represents a subfamily, and the number after the subfamily represents an individual gene [3]. All members within a family and a subfamily share >40% identity and >55% identity of their amino acid sequences, respectively [2]. To address the increasing number of P450 genes, higher-order groups have been proposed, with clans being defined as P450 families that are consistently clustered together in a phylogenetic tree, and clan names can be used to describe the higher-order evolution of gene superfamilies [3]. The insect P450 genes can be divided into four clans: the CYP2, CYP3, CYP4, and Mitochondrial (Mito) clans [3,4]. The current research on insect P450s mainly focuses on their metabolization of insecticides and plant secondary metabolites. Overexpression of P450 genes is an important mechanism for insect resistance to insecticides. *CYP6AE14* of the cotton bollworm *Helicoverpa armigera* can oxidize cotton phenols and its overexpression can also increase resistance to deltamethrin [5]. RNA interference (RNAi) of the *CYP6CM10* gene results in the reduced metabolization of neonicotinoid insecticides by the tobacco whitefly *Bemisia tabaci* [6]. It should not be ignored that P450s are also involved in catalyzing the biosynthesis and degradation of endogenous substances such as juvenile hormones (JH), ecdysteroids hormones (20E), fatty acids, etc., which are essential for the regulation of insects’ normal growth and development [7,8,9,10,11]. An important regulatory role in insects is played by 20E, participating in the regulation of insect growth, molting and metamorphosis, as well as in the regulation of adult behaviors, reproduction, lifespan, diapause, and innate immunity [12,13,14,15,16,17,18,19,20]. In studies on the fruit fly *Drosophila melanogaster*, at least five P450 genes were found to be involved in the synthesis of 20E [21]. The expression levels of *CYP307A1* and *CYP306A1* in the desert locust *Schistocerca gregaria* are related to the titer of 20E [22,23]. RNAi of *CYP314A1* in the diamondback moth *Plutella xylostella* can result in a delayed developmental cycle [24].

*Pseudoregma bambucicola* is a social aphid belonging to the tribe Cerataphidini of the subfamily Hormaphidinae. In most Asian subtropical areas of its distribution, *P. bambucicola* reproduces parthenogenetically all year on *Bambusa* hosts [25]. It can produce first-instar nymphs with morphological differentiation and functional division of labor within the colonies, namely normal nymphs and soldiers [26]. The soldiers can protect the colonies from enemies. Compared to the normal first-instar nymphs, the soldiers have sclerotized tergites, more enlarged forelegs, and longer and sharper frontal horns [27]. The soldiers are sterile and never develop into the second instar, while the first-instar normal nymphs can mature into adults with regular reproduction [26].

JH and 20E have been shown to play important roles in the regulation of social insect behavior and caste differentiation [28,29,30,31]. In terms of molecular mechanisms, the discovery of new genes and differential gene expression play an important role in the evolution of social insects [32,33,34,35,36]. In a previous study, *CYP6AM1* was found to affect the caste differentiation of the eusocial insect *Hodotermopsis sjostedti* [37]. In addition, P450 genes play an important role in the sociogenomics of social Hymenoptera [38]. However, the effect of P450 on social Hemiptera is not yet known. Considering that P450 genes are involved in the regulation of endocrine hormones, we hypothesized that P450 genes would be equally important for social insects in the Hemiptera. Thus, it is necessary to identify and analyze the P450 genes and their differential expression in different castes, which is important to understand whether different morphological characteristics, behaviors, and life history in *P. bambucicola* are related to P450 genes.

In this study, genome-wide identification and expression analysis of P450 genes were conducted based on the genome and transcriptome of *P. bambucicola*. Phylogenetic trees were constructed with other P450 genes of known function to inform the putative function of *P. bambucicola* P450 genes. The transcriptomic data were used to mine for highly expressed P450 genes in soldiers and to screen for candidate P450 genes responsible for the specific physiological phenomena of the soldiers.

## 2. Materials and Methods

### 2.1. Identification of P450 Genes in Pseudoregma bambucicola

Based on the genome project (GenBank BioProject ID: PRJNA913551) database of *P. bambucicola* constructed by the Insect Systematics and Diversity Lab at Fujian Agriculture and Forestry University, we used two methods to identify the P450 genes in *P. bambucicola.* Firstly, the hmmsearch program of HMMER3 (http://hmmer.janelia.org/, (accessed on 7 September 2022)) software was used to identify proteins with a P450 domain (PF00067, downloaded from http://pfam.xfam.org/, (accessed on 7 September 2022)) with an E-value < 10^−^^12^. Secondly, the P450 protein sequences of the pea aphid *Acyrthosiphon pisum* retrieved from the National Center for Biotechnology Information (NCBI) database were used to BLASTP against amino acid sequence databases of *P. bambucicola* via TBtools Blast (E-value < 10^−5^) to identify all possible P450 proteins in the *P. bambucicola* genome [39].

We then selected the intersection of the two sets of P450 proteins identified based on the two methods as the final result and used online FGENESH (http://linux1.softberry.com/berry.phtml, (accessed on 15 September 2022)) for structural correction of P450 genes without complete open reading frames. All candidate P450 genes were named by Dr. David Nelson (the Cytochrome P450 Nomenclature Committee) to maintain consistency in the nomenclature.

### 2.2. Bioinformatics Analysis

The average molecular weight and theoretical isoelectric point of each P450 gene were predicted by submitting sequences to ExPASy (http://web.expasy.org/protparam/, (accessed on 15 September 2022)). MEME (http://meme-suite.org/tools/meme, (accessed on 15 September 2022)) was used to predict the conserved motifs of each P450 gene. The graphics function in TBtools software [39] was used to extract the locus information of the P450 genes on chromosomes from the gff file of the gene annotation from the genome project mentioned above and plotted into a map. Adjacent genes separated by five or fewer genes were considered tandem gene duplications [40].

### 2.3. Comparison of P450 Gene Numbers between Pseudoregma bambucicola and Other Insects

The numbers of P450 genes from different insect species, including *A. pisum*, the green peach aphid *Myzus persicae*, the cotton aphid *Aphis gossypii*, the soybean aphid *Aphis glycines*, the silkworm *Bombyx mori*, *D. melanogaster*, *Anopheles gambiae*, the oriental fruit fly *Bactrocera dorsalis*, *Apis mellifera,* and the red flour beetle *Tribolium castaneum*, were collected through previous studies and the numbers of genes contained in each clan were listed in detail [41,42,43,44,45,46,47]. These species include a number of model insects and some closely related aphid species. The comparison of the number of P450 genes among different species provides a clearer reflection of the changes in the number of P450 genes in *P. bambucicola***.** This is a better way to explore whether P450 genes are specifically changed in *P. bambucicola*.

### 2.4. Phylogenetic Tree Reconstruction

PhyloSuite1.2.1 software [48] was used to reconstruct maximum likelihood phylogenetic trees of amino acid sequences of all the putative P450 genes in *P. bambucicola*, as well as all the P450 genes of *P. bambucicola* and the representative P450 genes of *A. pisum*, *A. gossypii*, *B. tabaci*, *D. melanogaster*, *B. mori,* and *A. mellifera*, respectively. The selected genes were those with well-studied functions and members belonging to similar families. Sequence alignments were performed using Muscle in MAGA5.2 software [49], and poorly aligned sequences from both ends were removed manually (alignments were provided with Data S1 and Data S2). The optimal models were tested by ModelFinder, and the LG+G4 model was selected for single-species tree reconstruction and the LG+F+R5 model for multi-species tree reconstruction. IQ-TREE integrated into PhyloSuite was used for maximum likelihood tree construction with 1000 bootstrapping replicates (see Data S3 and Data S4 for tree files).

The P450 gene sequences of these species were downloaded from the NCBI and Cytochrome P450 homepage (http://drnelson.uthsc.edu/CytochromeP450.html, accessed on 20 September 2022). See Table S1 for details.

### 2.5. Expression Analysis of P450 Genes

Transcriptome data sets of *P. bambucicola* across soldiers, first-instar nymphs, and adult viviparous females generated by our previous transcriptomic project (GenBank BioProject ID: PRJNA901050) were used for P450 gene expression analysis. The soldiers, first-instar nymphs, and adult aphids can be easily distinguished due to their distinct morphological differences. For transcriptome sequencing, 30 individuals were collected and prepared for each sample. The total RNA was extracted using the Trizol reagent method. The RNA concentration was determined using a NanoDrop 2000 spectrophotometer (Thermo Fisher Scientific, Waltham, MA, USA), and the RNA integrity was measured by using the RNA Nano 6000 assay kit. The qualified RNA was then used for cDNA library construction. The constructed cDNA libraries were sequenced by the Illumina HiSeq high-throughput sequencing platform. The obtained sequencing data were subjected to filtering adapter sequences, low-quality sequences, and ambiguous nucleotides (reads with more than 5% N bases) to obtain the final high-quality clean data that was used for de novo assembly with Trinity to obtain the final unigenes [50]. Bowtie was used for aligning clean reads to the unigene library and calculating the gene expression levels of unigene by using RSEM [51,52]. The expression abundance of the corresponding unigene was represented by the FPKM value (fragments per kilobase of exon model per million mapped fragments).

Differential expression analysis of the samples was conducted using the DESeq2 package [53]. Genes were assigned as differentially expressed with a threshold of false discovery rate (FDR) < 0.01 and a fold change ≥ 2 [54]. To investigate the P450 genes specifically expressed in soldiers, the expression levels of the P450 genes were then extracted and compared among soldiers, first-instar nymphs, and adult aphids.

## 3. Results

### 3.1. Identification of P450 Genes in Pseudoregma bambucicola

**Figure 1:**
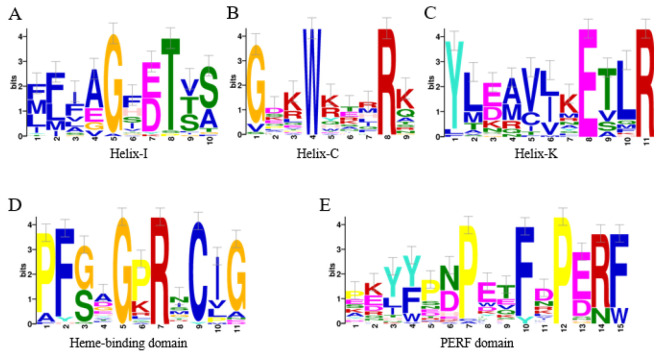
Motif and variation patterns among *Pseudoregma bambucicola* P450 genes. The height of a letter indicated its relative frequency at the given position for the amino acid. Small sample correction is shown above the amino acids. Five conserved regions are shown here: (**A**) helix-I (GxE/DTT/S); (**B**) helix-C (WxxxR); (**C**) helix-K (ExLR); (**D**) heme-binding domain (PFxxGxRxCxG/A); and (**E**) PERF domain (PxxFxPE/DR).

**Figure 2:**
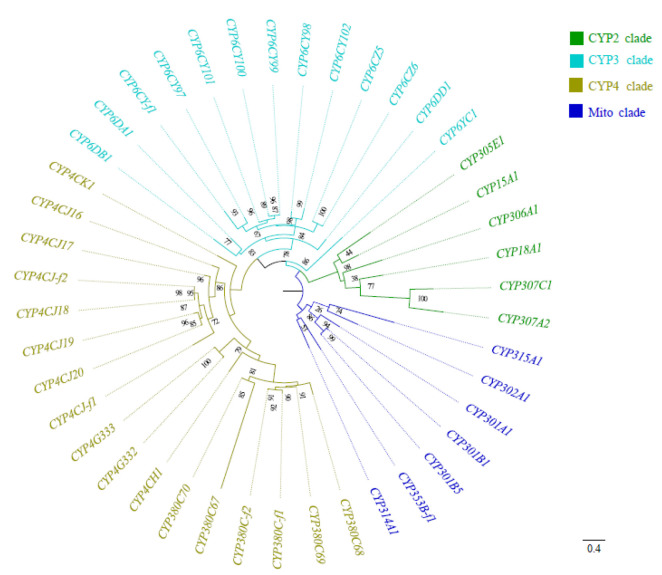
Phylogenetic analysis of P450 genes in *Pseudoregma bambucicola*. The tree was constructed with the maximum likelihood (ML) method using PhyloSuite. Bootstrap support values are indicated by numbers on nodes of phylogenetic tree. The clusters were highlighted in color to indicate different CYP clans. The letter “f” in the CYP name indicated that the P450 gene was a fragment.

In total, the same set of 43 cytochrome P450 genes was identified separately in the genome of *P. bambucicola* by 2 independent methods (Table 1). Among these 43 genes, 37 genes contained complete ORFs, and the 6 remaining genes were considered a partial sequence. The amino acid sequences of most of these P450s showed typical conserved P450 domains, including helix-C (WxxxR), helix-K (ExxR), helix-I (GxE/DTT/S), a PERF domain (PxxFxPE/DRF), and a heme-binding domain (PFxxGxRxCxG/A) (Figure 1). The amino acid residue lengths of the 43 P450 genes ranged from 131 aa to 840 aa, with molecular weights from 15.03 kDa to 96.95 kDa and isoelectric points from 5.39 to 10.14 (Table S2). These P450 genes were divided into CYP2, CYP3, CYP4, and Mitochondrial (Mito) clades (Figure 2), and the 4 clans were further classified into 13 families and 23 subfamilies (Table 1).

**Figure 3:**
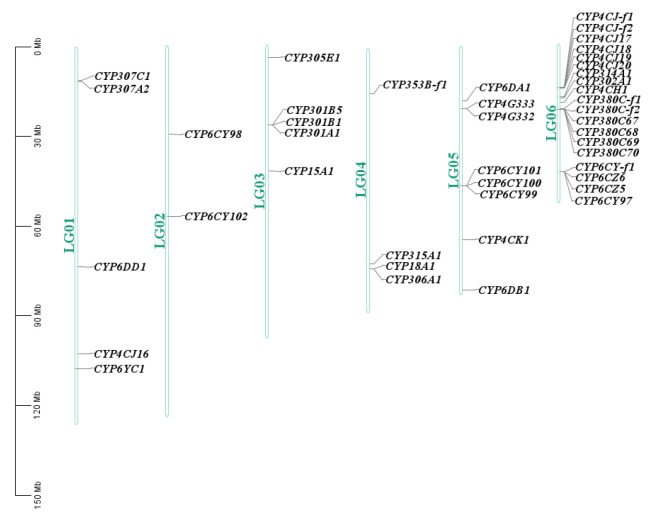
The chromosomal distribution of predicted P450 genes in the genome of *Pseudoregma bambucicola*.

We produced a high-quality map based on the *P. bambucicola* genome and located 43 P450 genes on 6 chromosomes/linkage groups (LGs) (Figure 3). In total, 22 of the 43 P450 genes were tandemly arranged into 5 clusters of at least 3 P450 genes. P450 genes are usually arranged in tandem, and this arrangement marks a P450 duplication event. The map reveals that the *P. bambucicola* P450 genes are unevenly distributed across the genome. For example, 20 P450 genes were located on chromosome 6. Chromosome 5 contains eight P450 genes. Similar numbers of genes were found on chromosomes 1, 3, and 4, which contained five, five, and four P450 genes, respectively. However, chromosome 2 only contained two P450 genes (Figure 3).

### 3.2. Comparison of P450 Genes between Pseudoregma bambucicola and Other Insects

The number of annotated P450 genes in *P. bambucicola* and other insect species from different orders (Coleoptera, Hymenoptera, Lepidoptera, Hemiptera, and Diptera) is listed in Table 2. The overall number of P450 genes in *P. bambucicola* is relatively small compared to most species. The CYP2 clan contained 6 genes, the CYP3 clan contained 13 genes, the CYP4 clan contained 17 genes, and the Mito clan contained 7 genes. Compared to other species, the number of P450 genes in the CYP3 clan was obviously reduced.

### 3.3. Phylogenetic Analysis of P450 Genes

**Figure 4:**
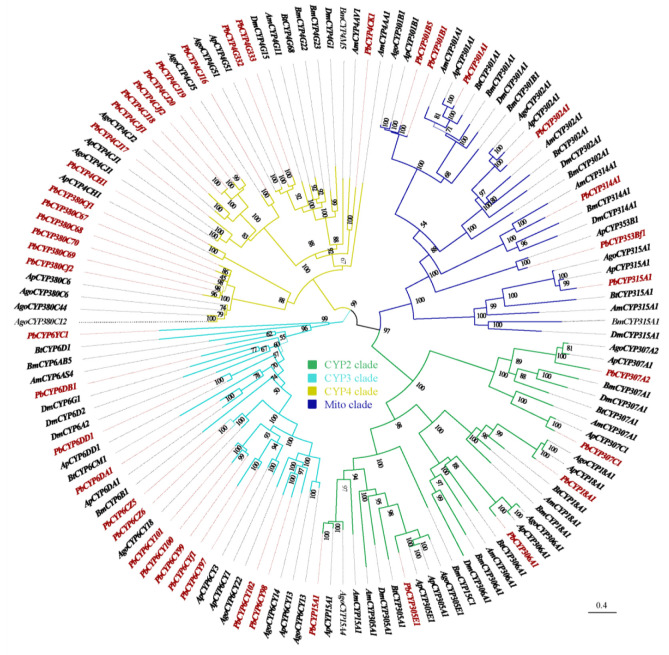
Phylogenetic relationship of P450 genes from *Pseudoregma bambucicola* (Pb), *Acyrthosiphon pisum* (Ap), *Aphis gossypii* (Ag), *Bemisia tabaci* (Bt), *Drosophila melanogaster* (Dm), *Bombyx mori* (Bm), and *Apis mellifera* (Am). Bootstrap support values are indicated by numbers on nodes of phylogenetic tree. The P450 genes of *Pseudoregma bambucicola* are marked in red in the figure.

The P450 genes of the *P. bambucicola* and six other insect species were selected to reconstruct a phylogenetic tree, which revealed the evolutionary relationships of P450 genes in these insects and provided insights into gene functions (Figure 4).

In *P. bambucicola*, the CYP4 clan was the largest clade, in which two families and five subfamilies were identified. Meanwhile, the CYP3 clan consisted of one family and six subfamilies. The CYP3 and CYP4 clans formed species-specific clusters within the phylogenetic tree. Compared with the CYP3 and CYP4 clans, the evolution and function of most P450 genes in CYP2 and Mito clans were considered to be highly conserved, leading to many families with few or even single genes. Most of the genes in these clades were found to exhibit a conserved 1:1 orthology on the phylogenetic tree.

### 3.4. Expression Pattern of P450 Genes in Pseudoregma bambucicola

**Figure 5:**
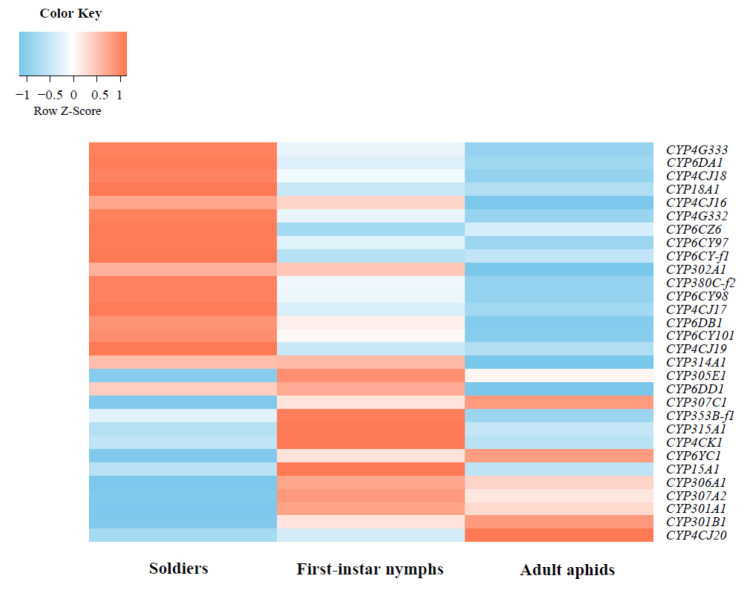
Expression patterns of P450 genes in soldiers, first-instar nymphs, and adult aphids of *Pseudoregma bambucicola*.

We analyzed the expression patterns of P450 genes in soldiers, first-instar nymphs, and adult aphids by using transcriptome data. Among these P450 genes, *CYP4G333*, *CYP4G332*, *CYP4CJ17*, *CYP6CY97*, *CYP6CY98*, *CYP6DA1*, and *CYP18A1* have higher levels of expression in soldiers compared with first-instar nymphs and adult aphids. We found that *CYP18A1* was obviously up-regulated (fold change ≥ 2) in soldiers compared with the first-instar nymphs and adult aphids. When compared with adult aphids, not only *CYP18A1*, but also *CYP4CJ16*, *CYP4CJ18*, *CYP4G333*, and *CYP6DA1* were up-regulated (fold change ≥ 2) in soldiers. In addition, *CYP306A1*, *CYP307A2*, *CYP301A1*, *CYP301B1,* and *CYP4CJ20* were all expressed at low levels in soldiers (Figure 5).

## 4. Discussions

The number of P450 genes in different insects varies greatly. In *P. bambucicola,* the number of P450 genes is 43, obviously smaller than in other insects. There may be three general reasons for this. Firstly, the technology of sequencing affects the number of P450 genes; for example, 35 and 34 P450 genes were identified based on two transcriptomic data sets of the cotton aphid *A. gossypii*, respectively [55,56]. In contrast, 49 P450 genes were identified in a study of the *A. gossypii* genome [42]. Our current data are based on the latest sequencing technology, which can reduce the effect of sequencing technology. Next, the highly organized true social nature of species may be partly responsible for the low number of P450 genes in the genome. For example, the eusocial *A. mellifera* also has a lower number of P450 genes (Table 2). Finally, changes in the number of genes in the CYP3 and CYP4 clans are now thought to be an adaptive strategy developed by insects in response to exogenous xenobiotics [57]. The number and variety of P450 genes associated with resistance metabolism are primarily strongly connected to the food source and habitat of insects [45,58]. There is a significant reduction in the numbers of both clans in the *P. bambucicola*, especially the CYP3 clan, which may be due to the fact that the *P. bambucicola* exclusively feeds on hard stems of bamboo.

In the CYP3 and CYP4 clans, genes from the same species cluster together to form paralog gene clusters that amplify heavily after species divergence, and this genetic requirement may be for insects to better cope with changes in the external environment. The *P. bambucicola* CYP3 clan includes one PbCYP6DB, one PbCYP6DD, seven PbCYP6CYs, two PbCYP6CZs, one PbCYP6DA, and one PbCYP6YC. Many studies suggest that the CYP6 family genes within the CYP3 clan are important for the detoxification of pesticides and the metabolism of plant secondary substances, enabling better adaptation of insects to host plants [59,60]. *CYP6CY3* in *M. persicae* can metabolize plant pyridine alkaloids and nicotine [61]. *CYP6CY14*, *CYP6DC1*, and *CYP6CZ1* can metabolize acetamiprid in *A. gossypii* [62]. Moreover, the CYP6CY subfamily is thought to be involved in the adaptation process of the soybean aphid *A. glycines* to plant hosts [63]. There is also experimental evidence that the *CPY6DA1* gene may be involved in the resistance of *A. gossypii* to the penetration of insecticides into the epidermis [64]. Gene duplication has occurred in the PbCYP6CY subfamily, which may be associated with the adaptation of *P. bambucicola* to a unique habitat. The exact function of these genes remains to be investigated, as even a single amino acid difference can alter substrate specificity [65].

Among *P. bambucicola* P450 gene clans, the CYP4 clan is the largest with five subfamilies, including six PbCYP380Cs, one PbCYP4CH, seven PbCYP4CJs, one PbCYP4CK, and two PbCYP4Gs. RNAi of the *CYP4G51* in the *A. pisum* leads to a decrease in cuticular hydrocarbon (CHC) content and desiccation tolerance, and in turn, increases mortality [66]. Additionally, RNAi of the *CYP4G1* gene in the fruit fly *D. melanogaster* resulted in a dramatic reduction in CHC content and a high sensitivity to desiccation stress [67]. Interference with genes of the CYP4G subfamily also leads to changes in the structure of the epidermis in other insect species, such as *Locusta migratoria* [68] and the brown planthopper *Nilaparvata lugens* [69]. Other functions have been identified in the ongoing study of the CYP4G subfamily. *CYP4G11* in *A. mellifera* is not only involved in the production of epidermal hydrocarbons but also functions to scavenge pheromones and phytochemical compounds from the antennae [70]. This suggests that the CYP4G subfamily encodes enzymes with a wide range of functions from desiccation resistance to chemical communication. In addition, *CYP4G1* in *D. melanogaster* and *CYP4G25* in *B. mori* were expressed in the prothorax, and *CYP4G25* was significantly highly expressed during diapause in *Antheraea yamamai* [71,72,73]. The above studies also support the notion that there must be one member of the CYP4G subfamily per insect involved in the biosynthesis of epidermal hydrocarbon [74].

*P. bambucicola* is a social insect with a high abundance of *CYP4G332* and *CYP4G333*, both of which are likely to play an important role in chemical communication. The tougher epidermis of soldiers may be associated with the significantly higher expression of *CYP4G333* in soldiers. The specific functions of these two genes still need to be further studied.

P450 genes belonging to the CYP380C subfamily have been previously implicated in the detoxification of xenobiotics. For example, *CYP380C6* and *CYP380C9* in *M. persicae* play a crucial role in mitigating indole glucosinolate-mediated plant defense [75], and the enzyme encoded by *CYP380C6* may contribute to detoxifying spirotetramat in *A. gossypii* [33]. From this aspect, the genes of the CYP380C subfamily of *P. bambucicola* are expressed and arranged in clusters, which may be better for the metabolization of phytochemicals and pesticides.

Genes from different species together form clusters of orthologous, which suggests that these genes are rarely amplified in these insects and have conserved functions [76]. The evolution of genes in the CYP2 and Mito clan are thought to be highly conserved, and many clades in both clans are found to consist of orthologous genes based on phylogenetic tree analysis, suggesting that these genes are conserved in function [77]. Previous studies have shown that P450 genes in these two clans are involved in the synthesis and metabolization of endogenous compounds in insects. For example, *CYP306A1* in the CYP2 clan and *CYP302A1*, *CYP314A1*, and *CYP315A1* in the Mito clan can encode enzymes that play important roles in the ecdysteroid synthesis pathway [78,79,80]. Although there is no *CYP307A1* in the *P. bambucicola*, the presence of the *CYP307A2* gene is found. This gene has been shown to catalyze the synthesis of ecdysteroid [81]. Decreased expression of *CYP307A2* leads to delayed development of *Laodelphax striatellus* and the tobacco whitefly *B. tabaci* [82,83]. The C26 hydroxylase encoded by *CYP18A1* converts 20E into a 26-hydroxylated metabolite through a hydroxylation reaction, and ultimately into the corresponding ecdysone acid. This is one of the key pathways of 20E metabolization [84,85,86]. Impaired expression of the *CYP18A1* gene in *D. melanogaster* leads to an increase in third-instar larvae and a lethal phenotype, while overexpression of the *CYP18A1* gene also shows lethal effects. In a study of *B. tabaci*, it was found that RNAi of *CYP18A1* expression delayed the life cycle of *B. tabaci* and resulted in a lethal phenotype [83]. The genes in the CYP2 clan have a high degree of amino acid similarity between different insect species and, therefore, can be used as a basis for speculating on similar functions [47]. Based on homology comparison, Pb*CYP18A1* was found to have a relatively high amino acid sequence homology with *D. melanogaster* (46.86%), *B. tabaci* (50.60%), and *A. gossypii* (90.24%). We hypothesize that Pb*CYP18A1* is conserved in function and capability to degrade 20E in vivo. *CYP15A1* is involved in the biosynthesis of insect JH and also shows a 1:1 orthology ratio between species [87,88,89]. *CYP301A1* has been described as an important gene involved in cuticle formation and may be involved in the regulation of 20E in this tissue [90].

Based on this evidence, the function of genes in the CYP2 and Mito clans are related to the biosynthesis or metabolization of endogenous compounds, which have an important role in the normal growth and development of insects. However, some genes in the CYP2 and Mito clans have also been found to have the ability to metabolize xenobiotics, which can help insects survive. For example, *CYP18A1* in the CYP2 clan could epoxidize aldrin to dieldrin and *CYP301B1* in the Mito clan enhances the resistance of *N. lugens* to plant-derived insecticide substances [77,91].

In *P. bambucicola, CYP306A1*, *CYP307A2,* and *CYP301A1* genes associated with 20E synthesis are significantly downregulated in soldiers compared to adult aphids. However, *CYP18A1*, a key gene for 20E metabolization, was significantly highly expressed in soldiers compared to first-instar nymphs and adult aphids. Therefore, we speculate that the expression of these genes may affect the titer of 20E in soldiers, which ultimately leads to soldiers’ sterility and failure to develop into the second instar.

Our study provides sequence information for 43 P450 genes from the *P. bambucicola* genome and analyzes the phylogenetic relationship with P450 genes from other insects. Expression analysis of P450 genes based on the *P. bambucicola* transcriptome data shows that *CYP4G333*, *CYP4G332*, *CYP4CJ17*, *CYP6CY97*, *CYP6CY98*, *CYP6DA1*, and *CYP18A1* have higher levels of expression in soldiers compared with first-instar nymphs and adult aphids. Among them, *CYP18A1* was significantly highly expressed in soldiers (fold change ≥ 2). In contrast to adult aphids, *CYP4CJ16*, *CYP4CJ18*, *CYP4G333*, and *CYP6DA1* were expressed at high levels (fold change ≥ 2). In addition, *CYP306A1*, *CYP307A2*, *CYP301A1*, *CYP301B1,* and *CYP4CJ20* were all expressed at low levels in soldiers. Differential expression of these genes may be one of the reasons for the soldiers’ hardened epidermis and arrested development. Other differences in gene expression exist and their specific functions need further study. This study may provide a basis for further exploring the function of P450 genes in *P. bambucicola* and provide useful information for the study of the behavior and development of *P. bambucicola*.

## Figures and Tables

**Table 1:** Family and subfamily of P450 genes in *Pseudoregma bambucicola*.

Clade	Family	Subfamily	Number of Genes
CYP2	305	305E	1
	15	15A	1
	306	306A	1
	18	18A	1
	307	307A, 307C	2
CYP3	6	6DB, 6DD, 6CY, 6CZ, 6DA, 6YC	13
CYP4	380	380C	6
	4	4CH, 4CJ, 4CK, 4G	11
Mitochondrial clade	353	353B	1
	301	301A, 301B	3
	302	302A	1
	314	314A	1
	315	315A	1

**Table 2:** Summary of P450s in *Pseudoregma bambucicola* and some other insect species.

	Species	CYP2	CYP3	CYP4	MitoCYP	Total
Hemiptera	*Acyrthosiphon pisum*	10	33	32	8	83
	*Myzus persicae*	3	63	48	1	115
	*Aphis gossypii*	9	20	16	4	49
	*Aphis glycines*	9	29	23	7	68
	*Pseudoregma bambucicola*	6	13	17	7	43
Lepidoptera	*Bombyx mori*	7	28	31	10	76
Diptera	*Drosophila melanogaster*	6	36	32	11	85
	*Anopheles gambiae*	10	42	45	9	106
	*Bactrocera dorsalis*	7	46	32	16	101
Hymenoptera	*Apis mellifera*	8	28	4	6	46
Coleoptera	*Tribolium castaneum*	8	79	47	9	143

## Data Availability

The genome and transcriptome data of *P. bambucicola* are available from NCBI under the BioProject numbers PRJNA913551 and PRJNA901050, respectively. The data presented in this study are available on request from the corresponding author.

## Supplementary Materials

The following supporting information can be downloaded at: https://www.mdpi.com/article/10.3390/insects14020212/s1, Table S1: acid sequences of P450 genes of *Pseudoregma bambucicola* and other insect species used for the phylogenetic analysis; Table S2: details of the 43 P450 genes identified in *Pseudoregma bambucicola*; Data S1: P450 gene sequence alignment file of *Pseudoregma bambucicola* for phylogenetic analysis; Data S2: P450 gene sequence alignment file of *Pseudoregma bambucicola* and other insect species for phylogenetic analysis; Data S3: phylogenetic tree file for P450 genes of *Pseudoregma bambucicola*; Data S4: phylogenetic tree file for P450 genes of *Pseudoregma bambucicola* and other insect species.
